# An explainable AI framework integrating machine and deep learning models for multi-species DNA functional group classification

**DOI:** 10.1038/s41598-026-53075-5

**Published:** 2026-05-18

**Authors:** Pratik Chakraborty, P. B. Shanthi

**Affiliations:** Manipal Institute of Technology, Manipal Academy of Higher Education, Manipal, India

**Keywords:** Attention heatmaps, DNA functional group classification, Deep learning, Explainable AI framework, k-mers, Machine learning, Healthcare AI, Bioinformatics, Computational biology and bioinformatics, Genetics

## Abstract

DNA functional group classification across species plays a crucial role in understanding genetic diversity, evolutionary relationships and biological function. The increasing availability of genomic data has led to the use of machine learning and deep learning methods for identifying functional patterns within DNA sequences. However, the interpretability of these models remains a challenge in validating biological relevance. This study presents an explainable AI framework that integrates machine learning and deep learning models for multi-species DNA functional group classification. The functional groups represent gene families, including transcription factors and kinases, and the classification task is carried out on Human, Chimpanzee, Dog, and a custom Combined dataset merging sequences from all three species. The DNA sequences were transformed into k-mers to capture local compositional patterns before training. Following a controlled hyperparameter tuning strategy, the Logistic Regression model consistently achieved the highest MCC and F1-scores across all evaluated datasets. While deep learning architectures captured longer motif dependencies, classical models showed stronger generalization across species. A multi-level XAI analysis was conducted using techniques such as Feature Importance, Saliency Maps, Integrated Gradients, GradientSHAP, and Attention Heatmaps. The analysis identified consensus motifs, cross-dataset and cross-model motif patterns, and evaluated model stability based on motif overlap and Jaccard similarity, as well as model fidelity based on performance drops after masking model-identified motifs.

## Introduction

A crucial aspect of understanding the processes of living organisms is the study of genomics. Genomics is a discipline that investigates the structure, function and regulation of genes across organisms. The discipline captures insights that help identify information encoded in DNA sequences^1^. Experimental methods, such as gene expression analysis using microarrays, have enabled simultaneous measurement of thousands of genes^2^. The systematic study of genes and genomes has further established frameworks for linking sequence-level data with functional interpretation^3^.

Genomics has many applications within the field of healthcare, ranging from diagnostics and personalized medicine^4^. These applications hold significant implications for disease prevention, early detection and the development of targeted treatments. A central focus of genomics lies in the analysis of DNA sequences, as variations and damage in DNA directly influence biological outcomes. For example, inherited disorders are often transmitted through defective DNA, where oxidative stress can damage germ-line cells, leading to infertility or childhood cancers^5^. Genes can be grouped into functional classes, such as G-protein coupled receptors and ion channels, each playing a critical role in maintaining biological processes. Although traditional sequence analysis methods have provided valuable insights, advances in AI-driven DNA classification now offer the potential for faster and more scalable identification of genetic risk factors^6^.

AI-driven approaches in multi-species DNA sequence classification have proven valuable for uncovering conserved genetic patterns, functional groups, and evolutionary relationships. Classical machine learning models such as Support Vector Machines (SVMs), Random Forests (RF), and Naive Bayes have been widely applied to tasks including alignment, classification, clustering and pattern discovery^7^. These models have also demonstrated practical utility in plant breeding by modeling large-scale SNP datasets^8^. Their ability to capture nonlinear relationships has facilitated the analysis of complex genomic signals. However, such models typically rely on handcrafted feature extraction, struggle with high-dimensional sequence data and often suffer from limited interpretability^9^.

Deep learning models offer key advantages over classical machine learning approaches, particularly by eliminating the need for handcrafted feature extraction. Architectures such as Convolutional Neural Networks (CNNs) and Recurrent Neural Networks (RNNs) can automatically learn discriminative features from raw sequence data and have been increasingly utilized for genome sequence analysis^9,10^. CNNs are highly effective in motif discovery and local feature extraction, while RNNs, especially Long Short-Term Memory (LSTM) networks and their bidirectional variants, excel at capturing long-range dependencies in sequence data. In short DNA sequence classification, CNN-based approaches have demonstrated accuracy comparable to alignment-based tools, while providing robustness to noisy and ambiguous reads^11^. In plant and animal breeding, deep learning has further proven powerful for modeling nonlinear genomic interactions given large datasets^12^. However, studies that identify functional gene families across multiple species are still limited, even though they are important for understanding shared mechanisms and unique adaptations.

Another crucial aspect shaping modern genomic research is the role of Explainable Artificial Intelligence (XAI). Various XAI techniques have been introduced and used in AI domains. Saliency maps introduced gradient-based explanations by highlighting the contribution of individual input features to model predictions^13^. Integrated Gradients addressed fundamental axiomatic gaps by ensuring sensitivity and implementation invariance in attributions^14^. SHAP provided a unified framework for feature attribution grounded in cooperative game theory, ensuring desirable properties such as local accuracy and consistency^15^. More recently, attention-based visual explanation methods, such as attention map-guided heatmaps, have further enhanced interpretability by localizing the most influential input regions with high class specificity^16^.

In genomics, XAI systems aim to improve transparency by revealing biological motifs and regulatory patterns hidden within the black-box predictions of AI models^17–20^. Techniques such as SHAP and Grad-CAM have been applied to uncover biologically meaningful features in genomic data, highlighting motifs and sequence patterns relevant to functional interpretation^21^. Beyond attribution techniques, transformer architectures have demonstrated the ability to specialize attention heads for detecting transcription factors and promoter elements in *E. coli*, thereby linking predictive performance with interpretable biological mechanisms^22^. XAI has also extended into applied domains such as forensic DNA profiling, where interpretable models help predict the number of contributors (NOC) from short tandem repeat (STR) mixtures, underscoring the expanding role of explainability across both genomic research and forensic contexts^23^.

While machine learning (ML), deep learning (DL), and explainable AI (XAI) have been applied to DNA sequence classification, there is limited work done on developing an explainable AI framework on DNA functional group classification that integrates various machine learning and deep learning models, along with multiple XAI techniques. Moreover, most existing works focus on a single species rather than exploring the performance of models on multi-species DNA sequences. To address these gaps, our study makes the following key contributions:

- We present an explainable AI framework that integrates machine and deep learning models for multi-species DNA functional group classification.
- We perform controlled hyperparameter tuning, robustness evaluation, and statistical significance testing for all models to ensure fair and consistent benchmarking.
- We apply multiple XAI techniques (Feature Importance, Saliency maps, Integrated Gradients, GradientSHAP and Attention heatmaps) to each model.
- We conduct a comprehensive XAI analysis that identifies consensus motifs and examines cross-dataset and cross-model motif patterns. The analysis also evaluates model stability using motif overlap and Jaccard similarity, and assesses model fidelity based on performance drops after masking model-identified motifs.
- We compare the method of our framework with previous studies on the same datasets to validate the framework.

The remainder of the paper is organized as follows: “Related work” reviews related work on DNA sequence classification, including machine learning, deep learning and explainable AI approaches. “Methodology” details the proposed explainable AI framework, including datasets, preprocessing techniques, model architectures, and interpretability methods. “Results and discussion” presents the experimental results and analyzes the classification performance, stability characteristics, and fidelity behaviour of the models, along with the biological relevance of the motifs uncovered through the multi-level XAI analysis. Finally, “Conclusion” concludes the paper and discusses future directions.

## Related work

DNA sequence classification has been extensively studied using a range of computational approaches, from traditional alignment-based methods to modern machine learning and deep learning frameworks. Recent work has outlined the strengths and limitations of classical ML models, such as SVMs and Random Forests, which rely on handcrafted features, as well as DL models like CNNs and RNNs that automatically extract sequence-level representations. More recently, XAI techniques have been introduced in genomics to enhance the interpretability of predictions and uncover biologically meaningful patterns. While extensive research has been conducted on single-species datasets, work on multi-species DNA classification remains limited, with only a few studies demonstrating its potential and leaving significant scope for systematic benchmarking. In this section, we review prior work under three themes: machine learning models, deep learning models and explainability techniques for DNA sequence classification.

### Machine learning approaches

Machine learning approaches for DNA sequence classification typically rely on handcrafted feature attributes such as k-mers, where DNA sequences are converted into feature vectors for classification^24^. A range of k values has been explored, with studies showing values between 5 and 7 to be particularly effective^25^. Research on human DNA sequences using models such as Random Forests and Naive Bayes has reported accuracies above 90%, demonstrating the effectiveness of these methods in single-species contexts^26,27^. Extensions to multi-species datasets, including dogs and chimpanzees, have achieved comparable accuracies for similar functional groups, highlighting the ability of ML models to generalize across species^28,29^.

Beyond standard classifiers, ML models have also been combined with metaheuristic and nature-inspired optimization algorithms such as Grey Wolf Optimization and Firefly Algorithm^30,31^. These methods enhance feature selection and model optimization, boosting classification performance in DNA sequence tasks^32^. Other studies have moved beyond sequence composition by incorporating structural and physical DNA features such as electrostatic potential, GC-content and DNA open-state energy^33^. Using these features, ML classifiers have achieved accuracies close to 90% in promoter prediction tasks.

These studies demonstrate the versatility of ML approaches across human and animal DNA sequences. However, systematic multi-species benchmarking of these ML models with rigorous hyperparameter tuning remains underexplored, which our paper addresses.

### Deep learning approaches

Deep learning (DL) models automatically learn features from DNA sequences, capturing a wide spectrum of sequence intricacies across longer spans. Approaches such as TF-IDF, one-hot encoding, and label encoding have been applied in conjunction with CNN, CNN-BiLSTM and other hybrid models for DNA sequence classification^34–36^. These methods, when optimized with genetic algorithms, have achieved accuracies as high as 94.88% for virus DNA classification^37^.

The advent of transformer models has further expanded applications of DL in genomics. Models such as Nucleic Transformer, DNABERT, and virusBERT employ stacked encoding and attention layers to capture sequence dependencies while providing added interpretability^38–40^. These models have been successfully applied to promoter detection, enhancer classification, and virus genome identification.

These works demonstrate the potential of both traditional DL architectures and transformer-based models for DNA sequence classification. However, their utilization for multi-species classification, combined with systematic hyperparameter tuning for robustness remains limited, which is a gap our paper addresses.

### XAI techniques

Explainable AI (XAI) techniques add a layer of interpretability to the black-box nature of AI models. Methods such as LIME and SHAP have been utilized with XGBoost for identifying COVID-19 gene biomarkers^41^. SHAP has also been applied with deep learning models such as CNNs and BERT for promoter identification and forensic DNA classification, thereby adding transparency to model predictions^42,43^. Beyond SHAP and LIME, Feature Importance methods have also been utilized to uncover crucial nucleotide positions such as A84, together with key sequence motifs^44^.

Other studies have introduced explainable neural frameworks that are inherently interpretable. ENNA (Explainable Neural Network Approximation), which integrates alignment-based and n-mer based classification through alignment trees, has been applied to DNA taxonomy classification^45^. Similarly, EDeepVPP and its hybrid variant extract viral genome patterns by learning the most important sequence filters^46^.

These works highlight the importance of incorporating trust and transparency in genomic AI models. However, limited work has been done on developing an explainable AI framework that integrates machine learning and deep learning models for multi-species DNA functional group classification, which is a gap our paper addresses.

## Methodology

This section details the comprehensive methodology for the paper. The proposed explainable AI framework integrates data preprocessing, ML and DL models, hyperparameter tuning, and XAI techniques to classify multi-species DNA functional groups.

**Fig. 1:**
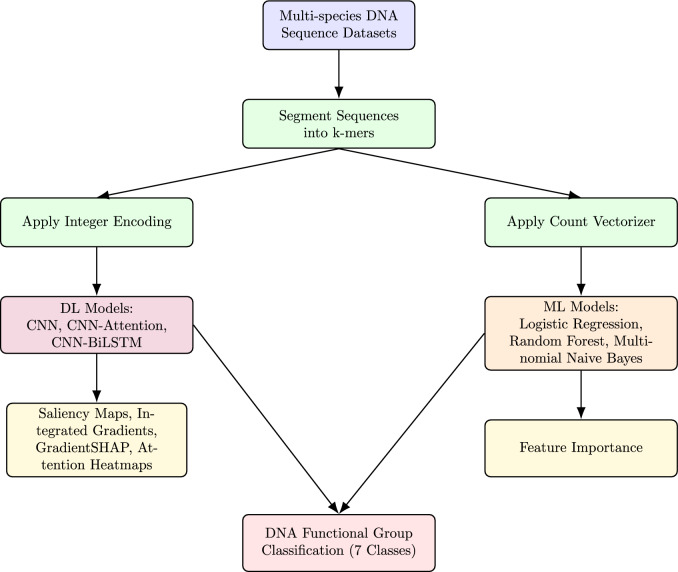
Proposed explainable AI framework for multi-species DNA functional group classification.

Figure 1 shows the proposed explainable AI framework for multi-species DNA functional group classification. The framework began with multi-species DNA sequence datasets, namely the Human, Chimpanzee, Dog, and Combined datasets, where the Combined dataset integrated sequences from all three species. Each dataset was partitioned using an 80:20 stratified train–test split, ensuring that class distributions were preserved across both sets. Furthermore, stratified five-fold cross-validation was applied to the training set during model development and hyperparameter tuning to ensure robustness and reduce bias.

Each dataset was segmented into k-mers, where k = 6. For ML models, the k-mers were converted into feature vectors using a Count Vectorizer approach. This approach was particularly suitable for ML algorithms as it provided sparse frequency-based representations that preserved token occurrence patterns effectively for classical classifiers. For DL models, the k-mers were encoded using integer encoding, which allowed sequences to be represented as index-based tokens, facilitating efficient embedding and learning of contextual patterns in DNA sequences.

The ML models employed included Logistic Regression, Random Forest, and Multinomial Naive Bayes, while the DL models consisted of CNN, CNN-Attention, and CNN-BiLSTM architectures. These models were trained on the training folds and evaluated on unseen DNA sequences from the held-out test set, which was not used during training or validation. In addition to predictive performance, explainability was incorporated into the proposed framework. For ML models, feature importance was applied to highlight the most influential k-mers in decision-making. For DL models, multiple explainable AI (XAI) techniques were integrated, including Saliency maps, Integrated Gradients, GradientSHAP, and attention heatmaps, to extract biologically meaningful motifs and provide interpretability alongside classification outcomes.

### Datasets used

Three publicly available DNA sequence datasets from Kaggle were utilized in this framework for multi-species DNA functional group classification^47^. In addition to the individual datasets, we constructed a combined dataset by merging all three sources. The datasets are denoted as Human, Chimpanzee and Dog, with a merged version referred to as Combined. Each dataset consists of DNA sequences for the respective species along with their corresponding functional group labels.

Table 1 summarizes the distribution of DNA functional groups across the Human, Chimpanzee, Dog, and Combined datasets. These DNA functional groups correspond to gene families which represent groups of proteins that share conserved sequence patterns, structural domains, and functional roles within cellular and regulatory processes. The Human dataset contains the largest number of samples (4380), followed by Chimpanzee (1682) and Dog (820). The Combined dataset therefore comprises 6882 sequences in total. These sample counts are important, as dataset size can directly influence the performance and stability of AI models during training and evaluation.

Although class imbalance is present, particularly in the Dog dataset, no resampling or reweighting techniques such as SMOTE or class weighting were applied, in order to preserve the original data distribution and ensure consistent evaluation across all models.

**Table 1:** Data distribution for DNA functional groups across Human, Chimpanzee, Dog, and the Combined dataset. The total count of DNA sequences for each dataset is also shown.

Functional group	Human	Chimpanzee	Dog	Combined
G protein-coupled receptors (GPCRs)	531	234	131	896
Tyrosine kinase	534	185	75	794
Tyrosine phosphatase	349	144	64	557
Synthetase	672	228	95	995
Synthase	711	261	135	1107
Ion channel	240	109	60	409
Transcription factor	1343	521	260	2124
Total	4380	1682	820	6882

Each functional group represents a biologically distinct class with important roles in cellular processes:

- G protein-coupled receptors (GPCRs): GPCRs are membrane proteins that detect extracellular signals and activate intracellular G proteins to regulate physiological processes such as vision, olfaction, and cardiovascular activity. They share a conserved seven-transmembrane structure with characteristic motifs including CWxP and DRY.
- Tyrosine kinases: Tyrosine kinases are enzymes that phosphorylate tyrosine residues on target proteins, controlling pathways involved in cell growth, differentiation, and immune signaling. They include receptor and non-receptor forms and contain conserved catalytic motifs such as the glycine-rich P-loop.
- Tyrosine phosphatases: Tyrosine phosphatases reverse kinase-mediated phosphorylation to maintain balanced signaling in pathways governing adhesion, migration, and immune responses. Their activity depends on the conserved HCX$_5$R catalytic motif, and dysregulation is linked to autoimmune and metabolic disorders.
- Synthetases: Aminoacyl-tRNA synthetases attach amino acids to their corresponding tRNAs, ensuring accurate translation. They contain well-conserved motifs such as HIGH and KMSKS and also perform additional regulatory roles in stress response and signaling.
- Synthases: Synthases catalyze biosynthetic reactions without requiring ATP hydrolysis, supporting key metabolic pathways including fatty acid and carbohydrate synthesis. Their modular structures enable efficient substrate binding and product formation.
- Ion channels: Ion channels are transmembrane proteins that control ion flow across membranes, regulating neuronal signaling, muscle contraction, and cardiac rhythm. They exhibit ion selectivity and diverse gating mechanisms, with dysfunction associated with neurological and cardiac diseases.
- Transcription factors: Transcription factors regulate gene expression by binding specific DNA motifs using structured domains such as zinc fingers or helix–turn–helix elements. They coordinate developmental programs and environmental responses, and disruptions in their function contribute to cancer and other disorders.

### Preprocessing steps

The preprocessing stage is a critical component of the proposed explainable AI framework, ensuring that raw DNA sequences are transformed into structured representations suitable for The processed sequences were then segmented into k-mers, where k represents the length of consecutive nucleotide substrings. Other techniques such as TF-IDF and one-hot encoding were not employed, as they do not effectively capture contiguous nucleotide relationships or motif structures, whereas k-mers explicitly model local sequence dependencies. In this study, a fixed value of k = 6 was used for all experiments. This choice aligns with common practice in DNA sequence analysis, where 6-mers provide an effective balance between computational efficiency and biological relevance. Six-nucleotide fragments capture meaningful local patterns, preserve short-range dependencies, and remain computationally tractable for large-scale modeling.

While longer biological motifs may span more than six nucleotides, the use of overlapping k-mers with a stride of one base ensures that extended sequence patterns are still represented as combinations of adjacent k-mers. This enables the models to capture longer-range dependencies implicitly through feature aggregation, rather than relying solely on a single fixed-length representation.

At the same time, adopting a fixed k-mer length introduces an inherent trade-off, as conserved motifs across different functional groups may vary in length. Consequently, a single k value may emphasize patterns aligned with the chosen k-mer size while providing a more uniform and consistent representation across datasets. To maintain comparability across models and ensure a controlled evaluation framework, this study employs a fixed k-mer setting.

The sequences were tokenized into overlapping 6-mers with a stride of one base, ensuring that every nucleotide position contributes to multiple contextual windows without information loss.

**Fig. 2:**
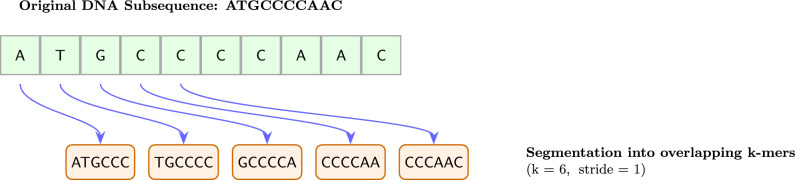
Illustration of DNA subsequence segmentation into overlapping k-mers for k = 6. Each k-mer represents a local substring of six nucleotides (ATGCCC, TGCCCC, GCCCCA, CCCCAA, and CCCAAC). The overlapping extraction ensures that every nucleotide contributes to multiple contextual windows.

Figure 2 illustrates this segmentation process, where a DNA subsequence is divided into overlapping k-mers to capture local context.

After segmentation, two distinct encoding strategies were applied depending on the downstream model architecture:

- Count vectorizer: For the machine learning (ML) models, the k-mers were converted into numerical feature vectors using the Count Vectorizer technique, which represents the frequency distribution of each unique k-mer across the dataset. The resulting sparse matrix captures the occurrence pattern of subsequences within each DNA sample, making it particularly effective for statistical models such as Naive Bayes, Logistic Regression, and Random Forests. To improve efficiency and reduce noise from rare patterns, the vocabulary was restricted to the most frequently occurring k-mers in the training set. The size of the feature vector is 4108, 4469, 4186, and 4556 for the Chimpanzee, Human, Dog, and Combined Dataset respectively.
- Integer encoding: For the deep learning (DL) models, each unique k-mer was assigned a distinct integer value to form a tokenized representation of the DNA sequence. This encoding preserves the sequential order of the nucleotides, allowing the models to learn both spatial and contextual relationships between adjacent motifs. The integer-encoded sequences were then padded or truncated to a uniform length to enable batch processing and stable gradient propagation during training. The size of the feature vector is 4108, 4469, 4186, and 4556 for the Chimpanzee, Human, Dog, and Combined Dataset respectively.

Alternative DNA sequence representations based on physicochemical properties or handcrafted feature extraction have been explored in prior work, however, such approaches introduce heterogeneous feature spaces that make direct comparison across ML and DL models more challenging. In this study, a k-mer-based representation is adopted to ensure consistency across models and to enable interpretable motif-level analysis within an explainable AI framework.

In addition to sequence encoding, the functional group labels were also integer-encoded to facilitate multi-class classification across both ML and DL models.

### Machine learning models used

**Fig. 3:**
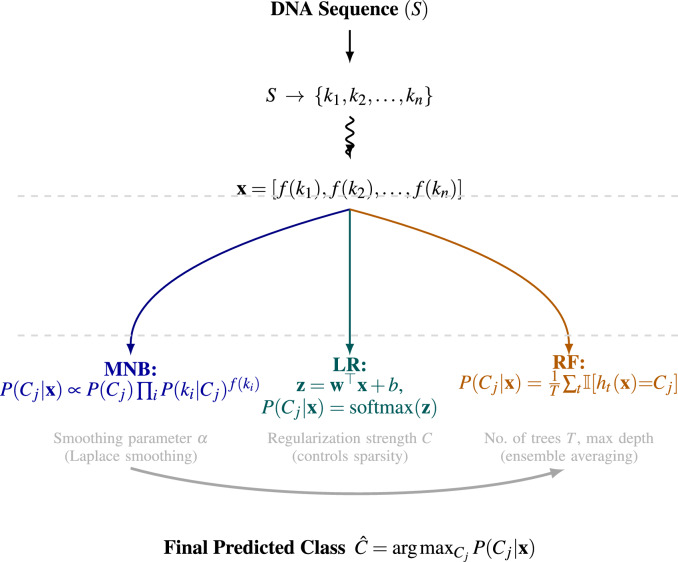
Unified conceptual overview of the machine learning branch. DNA sequences are segmented into overlapping k-mers and converted to frequency vectors using Count Vectorizer. These vectors are used as shared input to three models: MNB (probabilistic reasoning using Bayes’ theorem), LR (linear discriminative mapping via softmax regression), and RF (non-linear ensemble inference through decision-tree aggregation). Each path produces class probabilities $P(C_j|\textbf{x})$, culminating in the predicted functional class $\hat{C}$.

Within the ML branch of the explainable AI framework, three models were used: Multinomial Naive Bayes (MNB), Logistic Regression (LR), and Random Forest (RF). Models such as SVM were excluded because they are impractical for high-dimensional sparse k-mer feature spaces typical of DNA sequences, and MLPs were omitted because they offer lower interpretability for motif-level analysis compared to the selected models. Each of the three models captures different statistical and structural properties within the k-mer frequency distributions derived from DNA sequences. The unified conceptual workflow for these models is illustrated in Fig. 3.

MNB was chosen for its effectiveness in handling sparse k-mer count vectors and its probabilistic modeling of high-dimensional frequency data. Its Laplace smoothing parameter $\alpha$ provides robustness to rare or unseen k-mers while retaining sensitivity to discriminative motifs. LR was selected as an interpretable linear classifier that learns direct associations between k-mers and functional classes. The regularization parameter *C* controls model complexity, enabling strong generalization while preserving biologically meaningful feature weights. RF was incorporated to capture non-linear interactions among k-mers through an ensemble of randomized decision trees. Optimizing the number and depth of trees enhances predictive stability and provides reliable feature importance scores for extracting key biological patterns.

Collectively, these three ML models constitute the ML branch and serve as robust baselines for evaluating traditional approaches within the overall explainable AI architecture.

### Deep learning models used

**Fig. 4:**
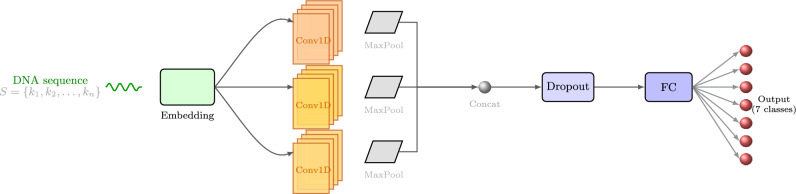
CNN architecture illustrating the three-branch convolutional feature extractor for DNA sequence classification. The embedded sequence is processed through parallel Conv1D layers that capture local motif patterns at different receptive field scales. The resulting feature maps are globally max-pooled, concatenated, regularized through dropout, and passed to a fully connected layer for final class prediction.

**Fig. 5:**
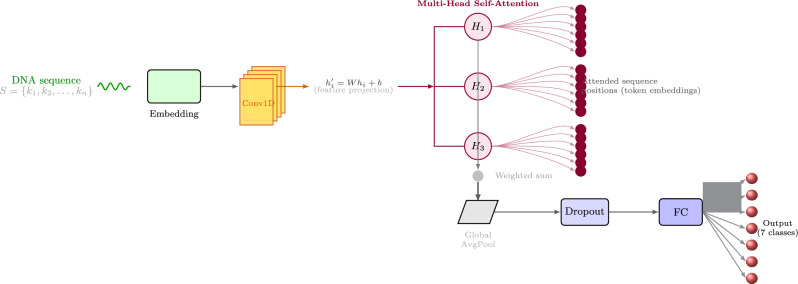
CNN–attention architecture illustrating the integration of convolutional motif embeddings with self-attention for modeling long-range dependencies in DNA sequences. The convolutional outputs are linearly projected ($h_i' = W h_i + b$) and processed by multiple attention heads to capture cross-motif interactions. The attended features are globally aggregated, regularized through dropout, and passed to a fully connected layer for final classification.

**Fig. 6:**
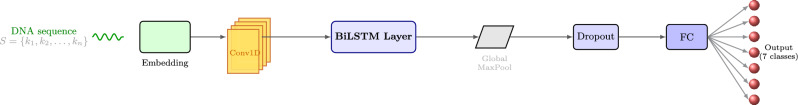
CNN–BiLSTM architecture illustrating the integration of convolutional motif extraction with bidirectional sequence modeling for DNA sequence classification. Local motif features learned through convolution are contextualized in both forward and reverse directions by the BiLSTM layer. The aggregated representations are globally max pooled, regularized through dropout, and passed to a fully connected layer for final prediction.

For the deep learning branch of the framework, integer-encoded k-mers were treated as sequential input tokens. Three architectures were implemented to capture both short-range motif patterns and long-range contextual dependencies in DNA sequences. The models, CNN, CNN-Attention, and CNN-BiLSTM, share the same high-level pipeline of embedding, feature extraction, and classification, differing primarily in how they model inter-k-mer relationships. Figures 4, 5, and 6 illustrate their respective architectures.

- Convolutional neural network (CNN): The CNN model was chosen for its ability to detect localized nucleotide motifs that strongly influence functional classification. It uses parallel one-dimensional convolutional filters with global max pooling and a dense output layer to capture multi-scale motif patterns.
- CNN-attention: The CNN-Attention model was selected to combine local motif extraction with global dependency modeling across distant sequence regions. It applies convolutional filters followed by multi-head self-attention and global pooling to learn both spatially local and context-aware representations.
- CNN-BiLSTM: The CNN-BiLSTM model was chosen to capture short-range motifs as well as directional and long-range dependencies within DNA sequences. Convolutional layers extract motif features that are processed by a bidirectional LSTM and pooled before classification to model sequential biological structure.

All deep learning models were trained using the Adam optimizer with categorical cross-entropy loss. Early stopping (patience = 5) was applied to prevent overfitting, with a maximum limit of 30 epochs. Padding ensured uniform sequence lengths across batches for stable GPU utilization. Dropout and learning-rate tuning were systematically performed for each model to balance generalization and convergence stability.

### XAI techniques used

The final stage of the proposed framework focuses on explainability, which provides interpretive insight into how the machine learning (ML) and deep learning (DL) models make classification decisions based on DNA sequence features. While accuracy metrics evaluate performance, explainable AI (XAI) techniques reveal why a model predicts a particular functional group by uncovering biologically meaningful motifs, conserved regions, and dependencies across species. The following five methods were applied in this study, each grounded in a distinct interpretive principle and mathematical formulation.

- Feature importance: Feature importance quantifies the relative contribution of each input feature to the model’s prediction. For linear models such as Logistic Regression, importance is directly proportional to the absolute weight magnitude $|w_i|$, where $w_i$ denotes the model coefficient corresponding to feature $x_i$. For ensemble models such as Random Forest, importance is derived from the average reduction in node impurity (using Gini or entropy criteria) when splitting on a particular feature:
  1 $$\begin{aligned} I(x_i) = \frac{1}{T} \sum _{t=1}^{T} \sum _{n \in N_t(x_i)} \frac{N_n}{N_t} \, \Delta \text {Impurity}(n) \end{aligned}$$
  where $T$ is the number of trees in the forest, $N_t$ is the total number of samples in tree $t$, $N_n$ is number of samples at node $n$, and $\Delta \text {Impurity}(n)$ is the decrease in impurity caused by splitting on feature $x_i$. This approach was used to identify high-importance k-mers corresponding to conserved genomic motifs such as CpG dinucleotides, promoter elements, and codon triplets that differentiate DNA functional groups.
- Saliency maps: Saliency maps visualize how sensitive the model’s output is to small perturbations in each input feature. For a given model score $S_c(x)$ corresponding to class $c$, the saliency for input feature $x_i$ is computed as:
  2 $$\begin{aligned} \text {Saliency}(x_i) = \left| \frac{\partial S_c(x)}{\partial x_i} \right| \end{aligned}$$
  Here, $\frac{\partial S_c(x)}{\partial x_i}$ represents the gradient of the output score with respect to input $x_i$. Larger gradients indicate positions or motifs that strongly influence the model’s prediction. This technique was chosen to reveal position-level contributions of nucleotides, highlighting regions such as transcription start sites or signal peptide motifs that drive class discrimination.
- Integrated gradients (IG): Integrated Gradients overcome the noise and gradient saturation issues of standard saliency methods by integrating gradients along a straight-line path between a baseline input $x'$ (e.g., zero or neutral embedding) and the actual input $x$:
  3 $$\begin{aligned} \text {IG}_i(x) = (x_i - x'_i) \int _{\alpha =0}^{1} \frac{\partial S_c(x' + \alpha (x - x'))}{\partial x_i} \, d\alpha \end{aligned}$$
  where $x_i$ is the actual feature value (e.g., encoded k-mer), $x'_i$ is the baseline feature, and $\alpha$ is a scalar interpolation coefficient. By accumulating the gradients along this continuous path, IG attributes the change in model output to specific k-mers or regions, yielding smoother and more biologically coherent motifs. This method was used to identify extended DNA motifs (e.g., GC-rich regulatory elements) that influence functional classification.
- GradientSHAP: GradientSHAP extends Integrated Gradients by incorporating the fairness concept from Shapley values, which estimate feature contributions by averaging over all possible input orderings. It approximates these contributions using stochastic sampling of baselines $x'$ from a distribution $\mathcal {B}$ and interpolation factors $\alpha \in [0,1]$:
  4 $$\begin{aligned} \text {GS}_i(x) = \mathbb {E}_{x' \sim \mathcal {B}, \, \alpha \sim U(0,1)} \left[ (x_i - x'_i) \frac{\partial S_c(x' + \alpha (x - x'))}{\partial x_i} \right] \end{aligned}$$
  where $\mathbb {E}$ denotes the expectation over sampled baselines, and $U(0,1)$ is the uniform distribution for $\alpha$. By averaging over multiple perturbed versions of the input, GradientSHAP produces smoother, more stable attributions that generalize across runs and species. It was employed in this study to ensure robustness and consistency of motif interpretations across multiple model architectures.
- Attention heatmaps: In attention-based networks, interpretability arises naturally from the attention mechanism, which assigns a relevance score to every pair of tokens (k-mers) in a sequence. The attention score between query vector $q_i$ and key vector $k_j$ is computed as:
  5 $$\begin{aligned} \text {Attention}(i,j) = \frac{e^{(q_i \cdot k_j) / \sqrt{d_k}}}{\sum _{m} e^{(q_i \cdot k_m) / \sqrt{d_k}}} \end{aligned}$$
  where $d_k$ is the dimensionality of the key vectors, $q_i$ and $k_j$ are the query and key embeddings for positions $i$ and $j$. The exponential term ensures positive, normalized scores akin to a softmax distribution. By visualizing the attention matrix as a heatmap, it becomes possible to identify long-range dependencies and interactions between distant motifs, such as co-regulated enhancer-promoter pairs or recurring binding sites, that are otherwise difficult to detect through convolution alone.

These XAI techniques are used for downstream analyses such as deriving consensus motifs for each functional group, performing cross-model and cross-dataset comparisons to identify selective and stable motifs, and conducting fidelity tests using normal DNA sequences and masked versions of model-identified motifs.

### Stability and fidelity metrics

In addition to the XAI techniques described above, we define quantitative metrics to evaluate the stability and fidelity of the extracted motifs.

Motif overlap: Given two models *A* and *B*, let $M_A$ and $M_B$ denote the sets of top-*k* motifs identified by each model. The motif overlap is defined as:

6 $$\begin{aligned} \text {Overlap}(A,B) = |M_A \cap M_B| \end{aligned}$$

Jaccard similarity: To measure stability between models, we compute the Jaccard similarity index:

7 $$\begin{aligned} J(A,B) = \frac{|M_A \cap M_B|}{|M_A \cup M_B|} \end{aligned}$$

Overlap percentage: To normalize overlap relative to the number of selected motifs, we define:

8 $$\begin{aligned} \text {Overlap }\% = \frac{|M_A \cap M_B|}{k} \times 100 \end{aligned}$$

Fidelity (masking-based evaluation): To evaluate the importance of identified motifs, each motif is masked in the original DNA sequence by replacing it with neutral tokens such as “N”. Let *X* denote the original input sequence and $X_{\text {mask}}$ the masked sequence. The fidelity is quantified as the drop in model performance:

9 $$\begin{aligned} \Delta \text {Metric} = \text {Metric}(X) - \text {Metric}(X_{\text {mask}}) \end{aligned}$$

where Metric corresponds to Accuracy, F1-score, MCC, or AUC. A larger performance drop indicates greater reliance of the model on the identified motifs.

## Results and discussion

This section presents the results obtained from the experiments conducted within the proposed explainable AI framework for multi-species DNA functional group classification. The evaluation includes ML and DL models applied to the Human, Chimpanzee, Dog, and Combined datasets. In analysing model performance across these datasets, several assessments were conducted: hyperparameter tuning for each model, robustness testing by averaging results over multiple random seeds, and statistical significance testing comparing the best-performing ML or DL model with the remaining models on each dataset.

### Performance of the machine learning models

To evaluate the performance of the machine learning (ML) models, we adopted a multi-stage evaluation strategy. The first stage involved controlled hyperparameter tuning, where each model was trained under a set of predefined configurations. Hyperparameter tuning is important to ensure that each model operates under reasonable and representative parameter settings that generalize well across datasets. Table 2 lists the configurations explored for each model. To maintain computational efficiency and ensure fair comparison across models, we limited the search to three distinct and representative configurations per model. This approach allows coverage of the parameter space while avoiding excessive model-specific optimization that could introduce bias in benchmarking. Hyperparameter selection was performed using a manual grid-based approach over a predefined search space, with configurations chosen to capture meaningful variations in model behavior.

**Table 2:** The hyperparameter configurations used for tuning the machine learning models.

Model	Configurations
Multinomial Naive Bayes (MNB)	Config 1: $\alpha = 0.1$ Config 2: $\alpha = 1.0$ Config 3: $\alpha = 5.0$
Logistic Regression (LR)	Config 1: $C = 0.1$ Config 2: $C = 1.0$ Config 3: $C = 10.0$
Random Forest (RF)	Config 1: 100 trees Config 2: 300 trees Config 3: 300 trees, max depth = 30

**Table 3:** Performance of the machine learning models under the different hyperparameter configurations across the four datasets.

Dataset	MNB	LR	RF
Chimpanzee	Config 1: MCC 0.5438, F1 0.6119, AUC 0.8508 Config 2: MCC 0.5448, F1 0.6129, AUC 0.8500 Config 3: MCC 0.5451, F1 0.6120, AUC 0.8493	Config 1: MCC 0.8767, F1 0.9016, AUC 0.9727 Config 2: MCC 0.8804, F1 0.9039, AUC 0.9719 Config 3: MCC 0.8768, F1 0.9017, AUC 0.9711	Config 1: MCC 0.7692, F1 0.8187, AUC 0.9510 Config 2: MCC 0.7827, F1 0.8320, AUC 0.9588 Config 3: MCC 0.7754, F1 0.8268, AUC 0.9598
Human	Config 1: MCC 0.5461, F1 0.6099, AUC 0.8476 Config 2: MCC 0.5438, F1 0.6080, AUC 0.8468 Config 3: MCC 0.5357, F1 0.6005, AUC 0.8452	Config 1: MCC 0.9063, F1 0.9181, AUC 0.9841 Config 2: MCC 0.9034, F1 0.9163, AUC 0.9834 Config 3: MCC 0.8950, F1 0.9083, AUC 0.9833	Config 1: MCC 0.8476, F1 0.8792, AUC 0.9849 Config 2: MCC 0.8441, F1 0.8793, AUC 0.9879 Config 3: MCC 0.8536, F1 0.8848, AUC 0.9902
Dog	Config 1: MCC 0.4710, F1 0.5332, AUC 0.8219 Config 2: MCC 0.4716, F1 0.5310, AUC 0.8260 Config 3: MCC 0.5239, F1 0.5818, AUC 0.8256	Config 1: MCC 0.6464, F1 0.6843, AUC 0.8800 Config 2: MCC 0.6458, F1 0.6832, AUC 0.8773 Config 3: MCC 0.6533, F1 0.6878, AUC 0.8762	Config 1: MCC 0.4345, F1 0.4726, AUC 0.8686 Config 2: MCC 0.4814, F1 0.4990, AUC 0.8947 Config 3: MCC 0.4814, F1 0.4990, AUC 0.8947
Combined	Config 1: MCC 0.6015, F1 0.6570, AUC 0.8769 Config 2: MCC 0.6006, F1 0.6567, AUC 0.8768 Config 3: MCC 0.6004, F1 0.6555, AUC 0.8766	Config 1: MCC 0.9244, F1 0.9379, AUC 0.9947 Config 2: MCC 0.9245, F1 0.9400, AUC 0.9938 Config 3: MCC 0.9227, F1 0.9375, AUC 0.9932	Config 1: MCC 0.8783, F1 0.9040, AUC 0.9881 Config 2: MCC 0.8854, F1 0.9102, AUC 0.9899 Config 3: MCC 0.8834, F1 0.9105, AUC 0.9900

Table 3 presents the performance of the ML models under the different hyperparameter configurations. Since the datasets are imbalanced, metrics such as MCC, F1-score, and AUC were used instead of accuracy, as they provide a more reliable and unbiased measure of performance. No single configuration consistently dominated across species for any model, underscoring the importance of systematic tuning, since it is not feasible to predict the best configuration for all datasets without rigorous empirical evaluation.

**Table 4:** Robustness evaluation of the tuned ML models by averaging results across five random seeds. The *p*-values represent the statistical significance of comparisons between the best-performing model and the alternatives.

Model	Human	Chimpanzee	Dog	Combined
Multinomial Naive Bayes	Accuracy: 0.6247 ± 0.0131 F1-score: 0.6211 ± 0.0119 MCC: 0.5617 ± 0.0135 AUC: 0.8691 ± 0.0127 p$_{\text {LR vs MNB}}$ = 4.44 $\times$ 10$^{-65}$	Accuracy: 0.5964 ± 0.0212 F1-score: 0.5899 ± 0.0205 MCC: 0.5285 ± 0.0229 AUC: 0.8472 ± 0.0153 p$_{\text {LR vs MNB}}$ = 3.52 $\times$ 10$^{-21}$	Accuracy: 0.5549 ± 0.0193 F1-score: 0.5205 ± 0.0240 MCC: 0.4651 ± 0.0241 AUC: 0.8107 ± 0.0306 p$_{\text {LR vs MNB}}$ = 0.0186	Accuracy: 0.6277 ± 0.0038 F1-score: 0.6282 ± 0.0062 MCC: 0.5670 ± 0.0049 AUC: 0.8638 ± 0.0073 p$_{\text {LR vs MNB}}$ = 1.62 $\times$ 10$^{-93}$
Logistic Regression	Accuracy: 0.9103 ± 0.0092 F1-score: 0.9050 ± 0.0129 MCC: 0.8901 ± 0.0114 AUC: 0.9847 ± 0.0023	Accuracy: 0.8991 ± 0.0145 F1-score: 0.9012 ± 0.0168 MCC: 0.8768 ± 0.0179 AUC: 0.9818 ± 0.0040	Accuracy: 0.7598 ± 0.0300 F1-score: 0.7389 ± 0.0246 MCC: 0.7029 ± 0.0380 AUC: 0.9251 ± 0.0219	Accuracy: 0.9467 ± 0.0054 F1-score: 0.9445 ± 0.0053 MCC: 0.9348 ± 0.0066 AUC: 0.9933 ± 0.0012
Random Forest	Accuracy: 0.8523 ± 0.0078 F1-score: 0.8544 ± 0.0140 MCC: 0.8252 ± 0.0095 AUC: 0.9841 ± 0.0040 p$_{\text {LR vs RF}}$ = 2.18$\times$10$^{-6}$	Accuracy: 0.8326 ± 0.0141 F1-score: 0.8425 ± 0.0191 MCC: 0.8008 ± 0.0163 AUC: 0.9727 ± 0.0079 p$_{\text {LR vs RF}}$ = 8.36$\times$10$^{-6}$	Accuracy: 0.6220 ± 0.0128 F1-score: 0.5937 ± 0.0307 MCC: 0.5341 ± 0.0174 AUC: 0.8896 ± 0.0188 p$_{\text {LR vs RF}}$ = 1.17$\times$10$^{-4}$	Accuracy: 0.9156 ± 0.0034 F1-score: 0.9216 ± 0.0038 MCC: 0.8987 ± 0.0041 AUC: 0.9938 ± 0.0006 p$_{\text {LR vs RF}}$ = 4.03$\times$10$^{-6}$

In the second stage, the best configuration for each model-dataset pair was evaluated for robustness. To ensure that performance was not dependent on a specific random seed, each tuned model was trained using five different random seeds, where each seed corresponds to a different stratified 80:20 train–test split. The evaluation metrics were averaged across these runs to obtain stable performance estimates.

We additionally performed statistical significance testing to assess whether the best-performing model on each dataset was meaningfully better than the alternatives. Specifically, McNemar’s test was applied on paired predictions, where predictions from all five seeds were concatenated to form a unified evaluation set. For each pair of models, a 2 $\times$ 2 contingency table was constructed based on agreement and disagreement in correct and incorrect predictions, and the exact version of McNemar’s test was used to compute the p-value. This approach ensures that statistical comparisons are performed on identical test instances across models.

The resulting p-values were consistently well below the standard significance threshold for most comparisons, indicating that the observed improvements are unlikely to be due to random variation. For comparisons with relatively larger p-values, results are interpreted conservatively. Table 4 reports these robustness evaluations.

Across all datasets, Logistic Regression achieved the strongest overall performance and reached a peak AUC of 0.9933 ± 0.0012 on the Combined dataset. Performance was lower on the Dog dataset due to its smaller size and high class imbalance, although Logistic Regression still maintained competitive results. Multinomial Naive Bayes underperformed because the k-mer distributions were not well aligned with its probabilistic assumptions, while Random Forest ranked second overall, benefiting from ensemble averaging but still trailing Logistic Regression on most datasets.

**Fig. 7:**
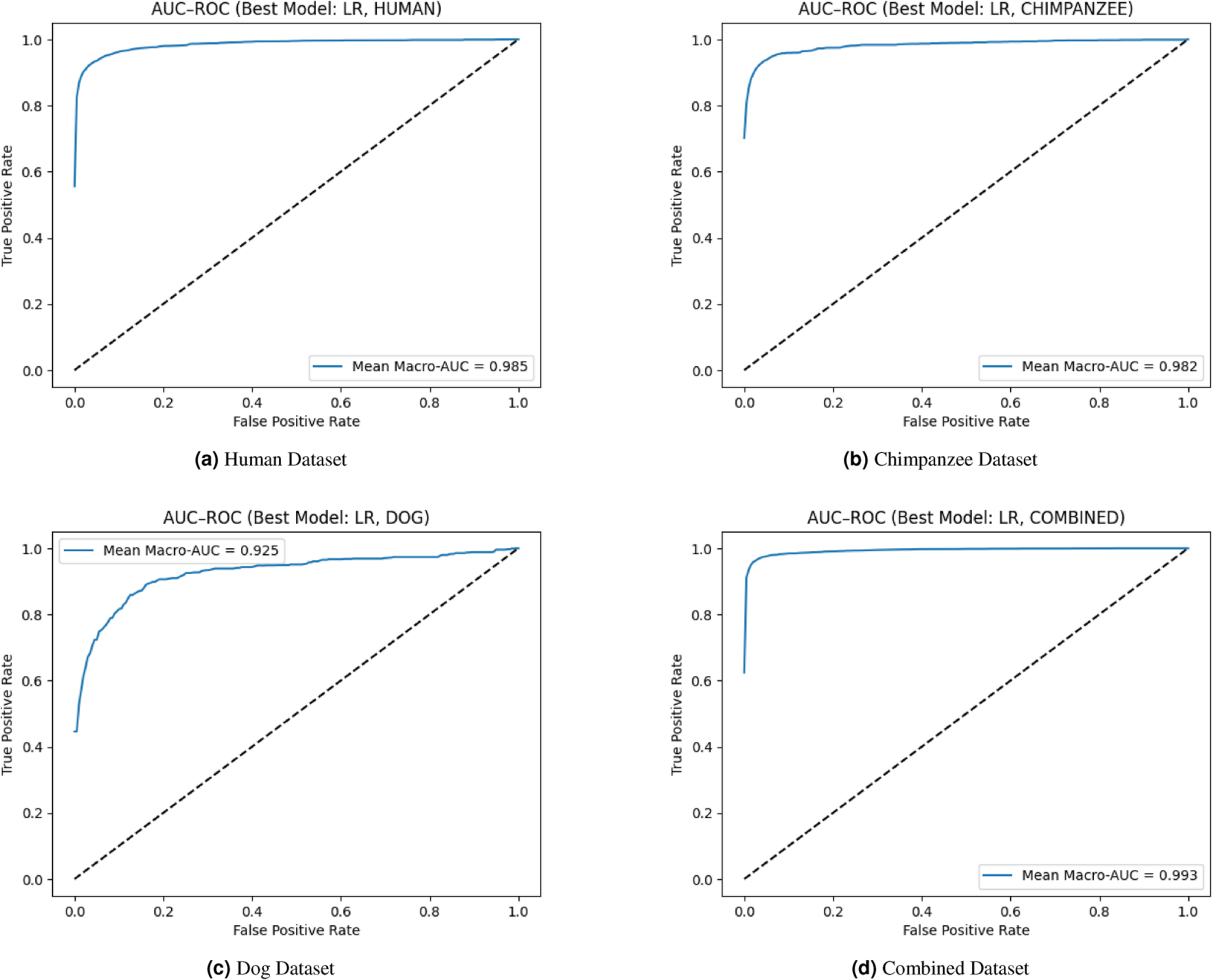
AUC–ROC curves of the best-performing ML model on each dataset.

For visual interpretation, Figure 7 presents the AUC–ROC curves of the best-performing model on each dataset. Because the task involves multiple classes, a one-versus-rest formulation was used, where each class was compared against all remaining classes. The curves show that the models were able to distinguish functional groups with high reliability, achieving near-perfect separability for most datasets. The Dog dataset exhibited the lowest performance, with an AUC of 0.925, reflecting the challenges posed by its limited size and imbalance.

### Performance of the deep learning models

The performance of the deep learning (DL) models was evaluated through the same multi-stage analysis applied to the ML models. Table 5 presents the hyperparameters considered for each DL architecture. These configurations were used during tuning, and each was evaluated on all four datasets. Hyperparameter optimization for the deep learning models was performed using a manual grid search over a predefined set of configurations, where a small but diverse search space was defined for each architecture. Each configuration was evaluated using a stratified 80:20 train–test split, ensuring consistent class distributions across training and test sets. To ensure fair comparison and robustness, the best-performing configuration for each model was further evaluated across five different random seeds, with results averaged to obtain stable performance estimates.

**Table 5:** The hyperparameter configurations used for tuning the deep learning models.

Model	Configurations
CNN	Config 1: embed_dim = 128, num_filters = 128, kernel_sizes = (3, 5, 7) Config 2: embed_dim = 128, num_filters = 256, kernel_sizes = (5, 7, 9) Config 3: embed_dim = 256, num_filters = 256, kernel_sizes = (3, 5, 7)
CNN–Attention	Config 1: embed_dim = 128, conv_filters = 128, conv_kernel = 7, n_heads = 2 Config 2: embed_dim = 128, conv_filters = 256, conv_kernel = 11, n_heads = 4 Config 3: embed_dim = 256, conv_filters = 256, conv_kernel = 15, n_heads = 4
CNN–BiLSTM	Config 1: embed_dim = 128, conv_filters = 128, lstm_hidden = 128 Config 2: embed_dim = 128, conv_filters = 256, lstm_hidden = 256 Config 3: embed_dim = 256, conv_filters = 256, lstm_hidden = 512

**Table 6:** Performance of the deep learning models under the different hyperparameter configurations across the four datasets.

Dataset	CNN	CNN–Attention	CNN–BiLSTM
Human	Config 1: ACC 0.9098, F1 0.9065, MCC 0.8897, AUC 0.9905 Config 2: ACC 0.9201, F1 0.9164, MCC 0.9027, AUC 0.9936 Config 3: ACC 0.9098, F1 0.9035, MCC 0.8898, AUC 0.9904	Config 1: ACC 0.8539, F1 0.8438, MCC 0.8215, AUC 0.9657 Config 2: ACC 0.8482, F1 0.8265, MCC 0.8159, AUC 0.9696 Config 3: ACC 0.8619, F1 0.8566, MCC 0.8312, AUC 0.9766	Config 1: ACC 0.9075, F1 0.9048, MCC 0.8873, AUC 0.9904 Config 2: ACC 0.9178, F1 0.9181, MCC 0.8995, AUC 0.9932 Config 3: ACC 0.9144, F1 0.9119, MCC 0.8953, AUC 0.9922
Chimpanzee	Config 1: ACC 0.8189, F1 0.8083, MCC 0.7798, AUC 0.9671 Config 2: ACC 0.8309, F1 0.8337, MCC 0.7969, AUC 0.9741 Config 3: ACC 0.8249, F1 0.8266, MCC 0.7864, AUC 0.9712	Config 1: ACC 0.7418, F1 0.7128, MCC 0.6830, AUC 0.9255 Config 2: ACC 0.7537, F1 0.7317, MCC 0.6978, AUC 0.9123 Config 3: ACC 0.8071, F1 0.7990, MCC 0.7638, AUC 0.9507	Config 1: ACC 0.8042, F1 0.7934, MCC 0.7637, AUC 0.9519 Config 2: ACC 0.7982, F1 0.8073, MCC 0.7591, AUC 0.9668 Config 3: ACC 0.8071, F1 0.8160, MCC 0.7690, AUC 0.9600
Dog	Config 1: ACC 0.4939, F1 0.3751, MCC 0.3649, AUC 0.7400 Config 2: ACC 0.4939, F1 0.3860, MCC 0.3817, AUC 0.7673 Config 3: ACC 0.5305, F1 0.4296, MCC 0.4411, AUC 0.7849	Config 1: ACC 0.4939, F1 0.3738, MCC 0.3578, AUC 0.6976 Config 2: ACC 0.4695, F1 0.3460, MCC 0.3346, AUC 0.7315 Config 3: ACC 0.4390, F1 0.3473, MCC 0.2921, AUC 0.7193	Config 1: ACC 0.5061, F1 0.4201, MCC 0.3782, AUC 0.7548 Config 2: ACC 0.4756, F1 0.3568, MCC 0.3738, AUC 0.7707 Config 3: ACC 0.5061, F1 0.4069, MCC 0.4127, AUC 0.8128
Combined	Config 1: ACC 0.9245, F1 0.9206, MCC 0.9078, AUC 0.9944 Config 2: ACC 0.9296, F1 0.9274, MCC 0.9143, AUC 0.9944 Config 3: ACC 0.9172, F1 0.9086, MCC 0.8995, AUC 0.9938	Config 1: ACC 0.8635, F1 0.8549, MCC 0.8328, AUC 0.9723 Config 2: ACC 0.8627, F1 0.8485, MCC 0.8323, AUC 0.9754 Config 3: ACC 0.8562, F1 0.8438, MCC 0.8251, AUC 0.9788	Config 1: ACC 0.9216, F1 0.9205, MCC 0.9042, AUC 0.9926 Config 2: ACC 0.9223, F1 0.9199, MCC 0.9053, AUC 0.9938 Config 3: ACC 0.9187, F1 0.9147, MCC 0.9008, AUC 0.9943

Table 6 reports the tuning results, where, similar to the ML case, no single configuration consistently dominated across datasets, reinforcing the need for dataset-specific hyperparameter exploration.

**Table 7:** Robustness evaluation of the tuned DL models by averaging results across five random seeds. The *p*-values represent the statistical significance of comparisons between the best-performing model and the alternatives.

Model	Human	Chimpanzee	Dog	Combined
CNN	Accuracy: 0.9059 ± 0.0079 F1-score: 0.9075 ± 0.0071 MCC: 0.8862 ± 0.0089 AUC: 0.9907 ± 0.0016	Accuracy: 0.8320 ± 0.0162 F1-score: 0.8364 ± 0.0162 MCC: 0.7989 ± 0.0176 AUC: 0.9698 ± 0.0077	Accuracy: 0.5768 ± 0.0224 F1-score: 0.5339 ± 0.0337 MCC: 0.4843 ± 0.0378 AUC: 0.8324 ± 0.0281 p = 0.8126	Accuracy: 0.9359 ± 0.0020 F1-score: 0.9331 ± 0.0035 MCC: 0.9219 ± 0.0023 AUC: 0.9964 ± 0.0003 p = 0.2313
CNN–Attention	Accuracy: 0.8550 ± 0.0085 F1-score: 0.8451 ± 0.0135 MCC: 0.8230 ± 0.0107 AUC: 0.9732 ± 0.0046 p = 1.30$\times$10$^{-30}$	Accuracy: 0.7780 ± 0.0238 F1-score: 0.7630 ± 0.0310 MCC: 0.7303 ± 0.0284 AUC: 0.9415 ± 0.0117 p = 1.07$\times$10$^{-11}$	Accuracy: 0.5085 ± 0.0099 F1-score: 0.4600 ± 0.0276 MCC: 0.3857 ± 0.0132 AUC: 0.7582 ± 0.0197 p = 0.00012	Accuracy: 0.8809 ± 0.0042 F1-score: 0.8713 ± 0.0062 MCC: 0.8546 ± 0.0048 AUC: 0.9783 ± 0.0016 p = 6.72$\times$10$^{-68}$
CNN–BiLSTM	Accuracy: 0.9014 ± 0.0043 F1-score: 0.9041 ± 0.0079 MCC: 0.8802 ± 0.0056 AUC: 0.9898 ± 0.0015 p = 0.1495	Accuracy: 0.8184 ± 0.0145 F1-score: 0.8277 ± 0.0148 MCC: 0.7826 ± 0.0174 AUC: 0.9655 ± 0.0048 p = 0.0235	Accuracy: 0.5732 ± 0.0332 F1-score: 0.5225 ± 0.0480 MCC: 0.4891 ± 0.0352 AUC: 0.8113 ± 0.0309	Accuracy: 0.9389 ± 0.0038 F1-score: 0.9399 ± 0.0039 MCC: 0.9255 ± 0.0045 AUC: 0.9962 ± 0.0004

Table 7 presents the robustness evaluation results using the best configuration per model. Among the architectures, CNN achieved the strongest performance on the Human and Chimpanzee datasets, whereas CNN–BiLSTM performed best on the Dog and Combined datasets. MCC was used as the primary evaluation metric due to its suitability for imbalanced multi-class settings, providing a balanced measure even when class frequencies differ substantially. Statistical significance testing using McNemar’s test showed that most pairwise comparisons yielded extremely low *p*-values, indicating that performance differences between the best model and the alternatives were statistically meaningful.

Although statistical significance testing was primarily reported within model families (ML and DL), all models were evaluated on identical test instances, allowing the same McNemar’s test framework to be extended for cross-family comparisons between Logistic Regression and deep learning models. This suggests that the observed performance gap between Logistic Regression and DL models is unlikely to be due to random variation.

Exceptions occurred for CNN and CNN–BiLSTM on the Dog and Combined datasets, where *p*-values ranged from 0.2 to 0.8, indicating that their performance was statistically comparable on these datasets. CNN performed well due to its strong ability to extract local motif-level patterns, while CNN–BiLSTM benefited from its capacity to capture both spatial and short-range sequential dependencies. In contrast, CNN–Attention underperformed relative to the other models, likely because attention mechanisms introduce additional model complexity and parameters and typically require larger datasets to learn stable and meaningful attention weights. In relatively smaller datasets, such as the Dog dataset, this can lead to less reliable attention distributions and reduced performance.

**Fig. 8:**
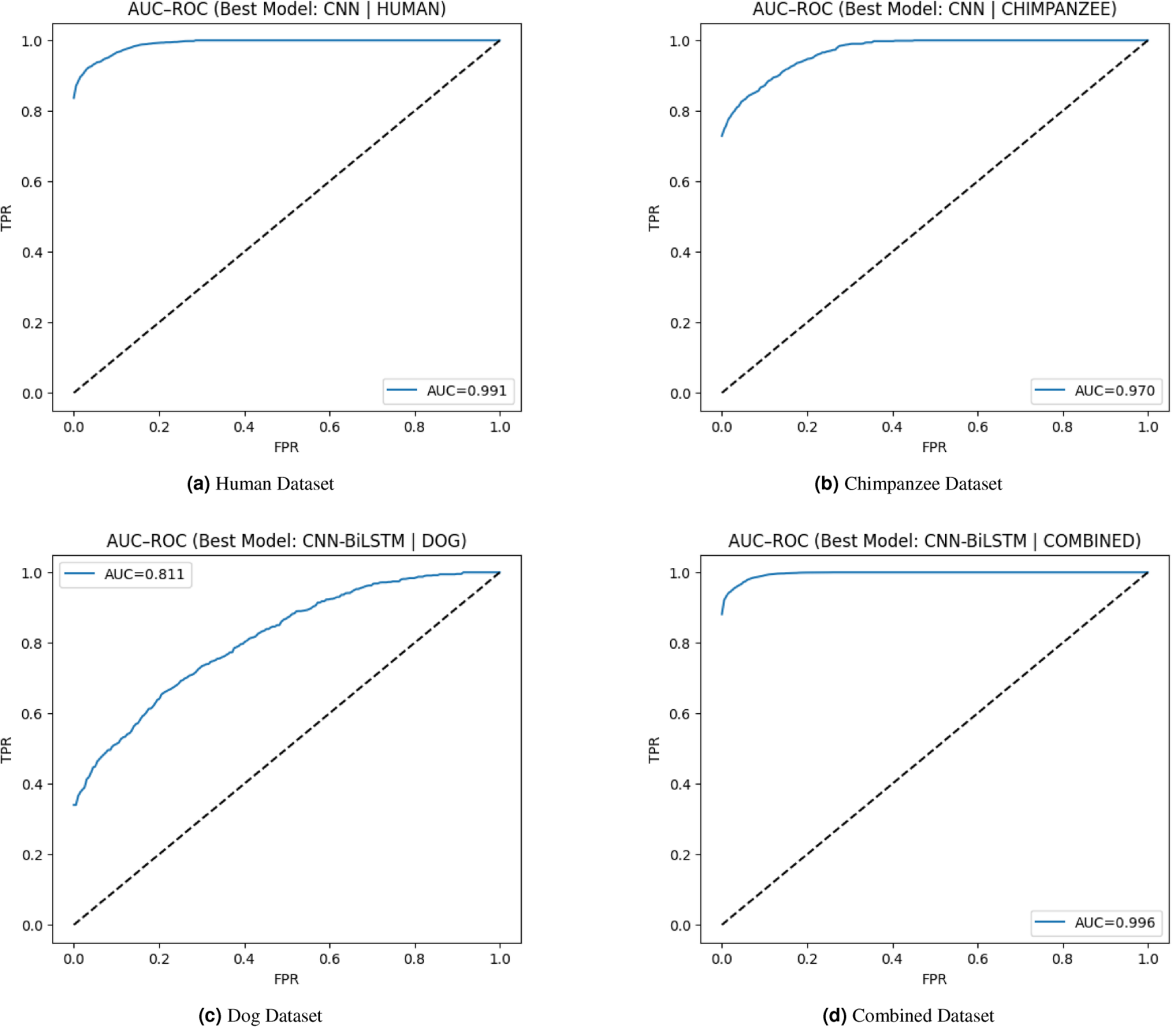
AUC–ROC curves of the best-performing DL model on each dataset.

Figure 8 shows the AUC–ROC curves for the best-performing DL model on each dataset. Similar to the ML results, the models achieved near-perfect separability for the Human, Chimpanzee, and Combined datasets. The Dog dataset exhibited a substantially lower AUC of 0.811, reflecting the challenges posed by severe class imbalance and limited sample size.

**Fig. 9:**
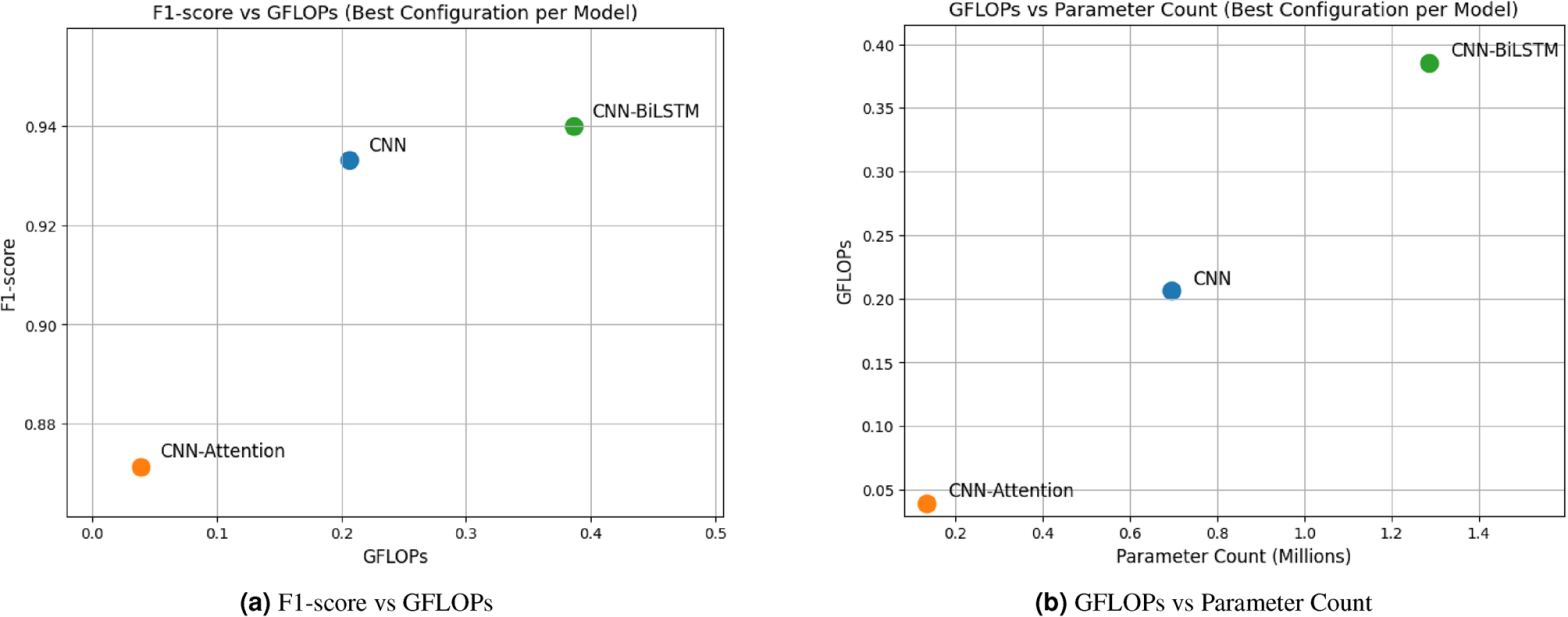
Model efficiency comparison across the deep learning models: (**a**) F1-score vs. GFLOPs, (**b**) GFLOPs vs. parameter count.

We additionally analyzed the computational efficiency of the DL models relative to their predictive performance. Figure 9a plots the F1-score against GFLOPs for the best configuration of each model (defined as the highest-performing instance across all datasets). CNN and CNN–BiLSTM occupy the upper-right region of the plot, indicating that they achieve strong performance at a moderate computational cost, whereas CNN–Attention lies significantly lower in both predictive ability and GFLOPs. Figure 9b further compares GFLOPs with parameter count. CNN–BiLSTM is the most parameter-heavy model and also exhibits the highest computational load, whereas CNN remains comparatively lightweight with a strong efficiency–performance trade-off. Collectively, these results show that CNN balances accuracy and efficiency most effectively, while CNN–BiLSTM provides higher representational capacity at the cost of increased computational complexity.

### Benchmarking against prior studies

**Table 8:** Comparison of prior studies on DNA functional group classification with the proposed method.

Study	Datasets used	Method
Juneja et al. ^25^ (2022)	Human, Chimpanzee, Dog	Used Multinomial Naive Bayes for functional group classification, achieving peak accuracies of 98.40% (Human), 91.40% (Chimpanzee), and 69.50% (Dog)
Pandya et al. ^28^ (2024)	Human, Chimpanzee	Applied multiple machine learning algorithms, reporting peak accuracies of 99% (Human) and 92% (Chimpanzee)
Jeslin et al. ^26^ (2025)	Human	Evaluated several ML algorithms, achieving a peak accuracy of 97% on the Human dataset
Pai et al. ^27^ (2025)	Human	Used multiple ML algorithms, including an optimized Multinomial Naive Bayes model, achieving a peak accuracy of 98% on the Human dataset
Proposed method	Human, Chimpanzee, Dog	Introduces an explainable AI framework that integrates multiple ML and DL models with robust hyperparameter tuning and a comprehensive multi-level XAI analysis, including cross-model, cross-dataset, and consensus motif evaluation for multiple DNA functional groups

We also compared our proposed framework with prior studies that used the same species-specific DNA datasets for functional group classification. Table 8 summarizes these methods, most of which relied solely on traditional machine learning models and evaluated performance primarily at the accuracy level. These approaches did not incorporate deep learning architectures, systematic hyperparameter tuning, or any form of explainability analysis, thereby limiting their ability to provide biologically interpretable insights.

In contrast, the novelty of our framework lies not only in integrating ML and DL models, but in introducing a structured, multi-level explainability pipeline that extends beyond standard feature attribution. Specifically, the combination of multiple XAI techniques enables consensus motif extraction, cross-model and cross-dataset comparative analysis, stability evaluation using Jaccard similarity, and fidelity assessment through motif masking experiments. This transforms the task from purely predictive modeling into interpretable pattern discovery, allowing us to analyze the consistency, robustness, and biological plausibility of learned sequence motifs. In addition to interpretability, our evaluation revealed that Logistic Regression achieved the highest MCC and F1-scores across all datasets when evaluated using averaged results over multiple runs, with particularly strong and stable performance across both individual and combined datasets. This highlights that, despite the use of more complex deep learning architectures, simpler linear models can remain highly effective for k-mer based DNA classification under controlled and reproducible evaluation settings. To the best of our knowledge, such a comprehensive and systematically designed XAI framework for DNA functional group classification has not been explored in prior work, thereby advancing current research beyond conventional performance-focused approaches.

### Explainability based motif analysis

A crucial component of the proposed framework is the explainable AI (XAI) analysis, which was applied to understand the motifs identified by the models for each functional group. We employed a multi-level XAI strategy to extract both model-specific and biologically meaningful insights. First, we identified consensus motifs that appeared consistently across models, datasets, and XAI techniques for each functional group and visualized them using bar charts. Next, we performed cross-species and cross-dataset comparative analysis for both ML and DL models to examine the most important motifs associated with specific functional groups. We then conducted a stability analysis, computing the overlap and Jaccard index across different ML–ML and DL–DL model pairs on the Combined dataset to measure the consistency of motif identification. Finally, we performed a fidelity analysis by masking the motifs identified as important and quantifying the resulting change in model performance, allowing us to assess the true reliance of each model on the extracted motifs.

**Fig. 10:**
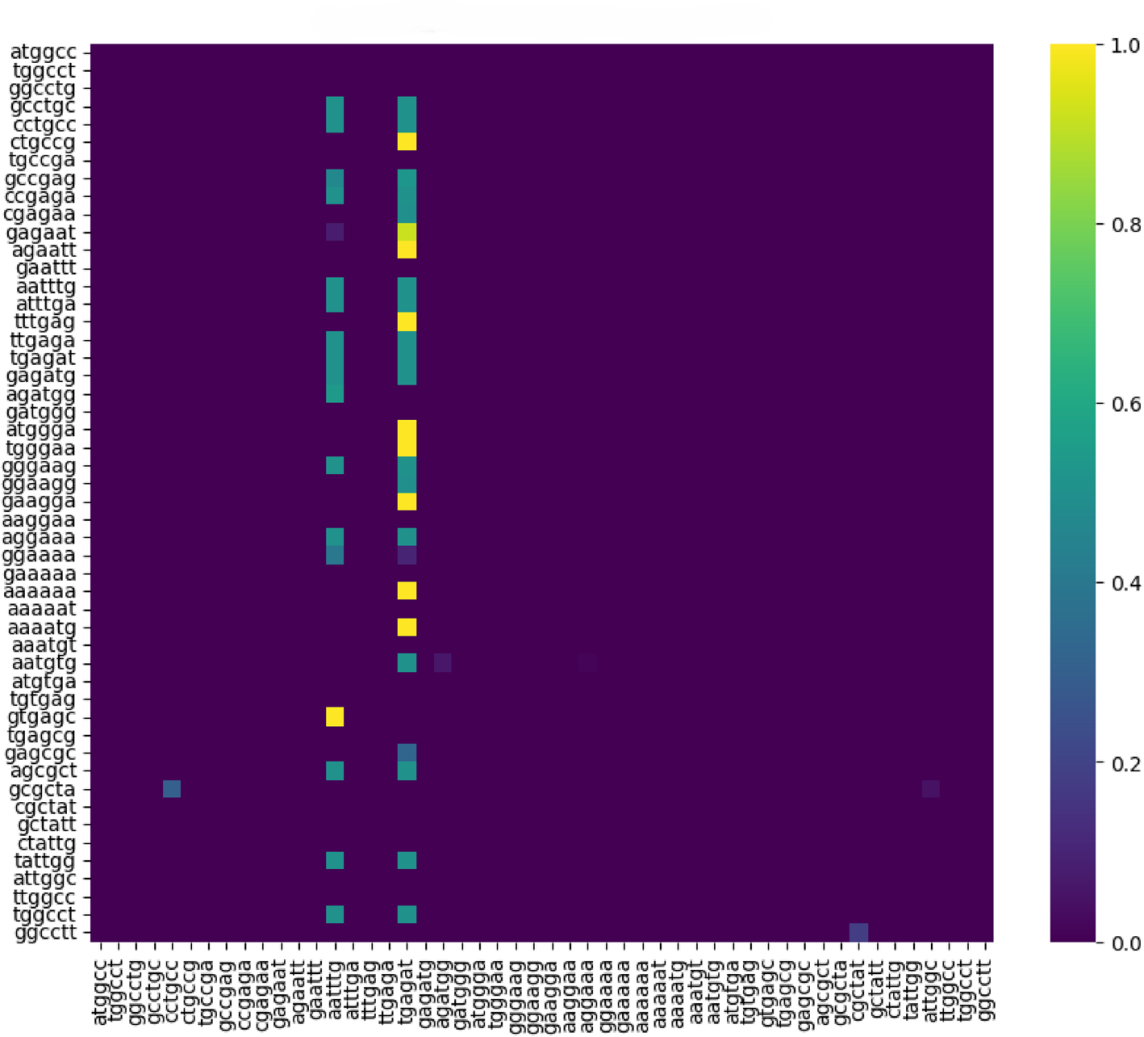
Attention heatmap for the CNN-Attention model on Tyrosine Phosphatase sequences. Brighter regions indicate stronger associations between motifs, whereas darker regions represent weaker contextual influence.

An Attention heatmap, illustrated in Fig. 10, shows how the CNN-Attention model assigns importance to specific motif pairs while predicting the Tyrosine Phosphatase group. Distinct high intensity regions correspond to interactions between k-mers that exert the greatest influence on the model’s decision.

#### Consensus motifs analysis

**Fig. 11:**
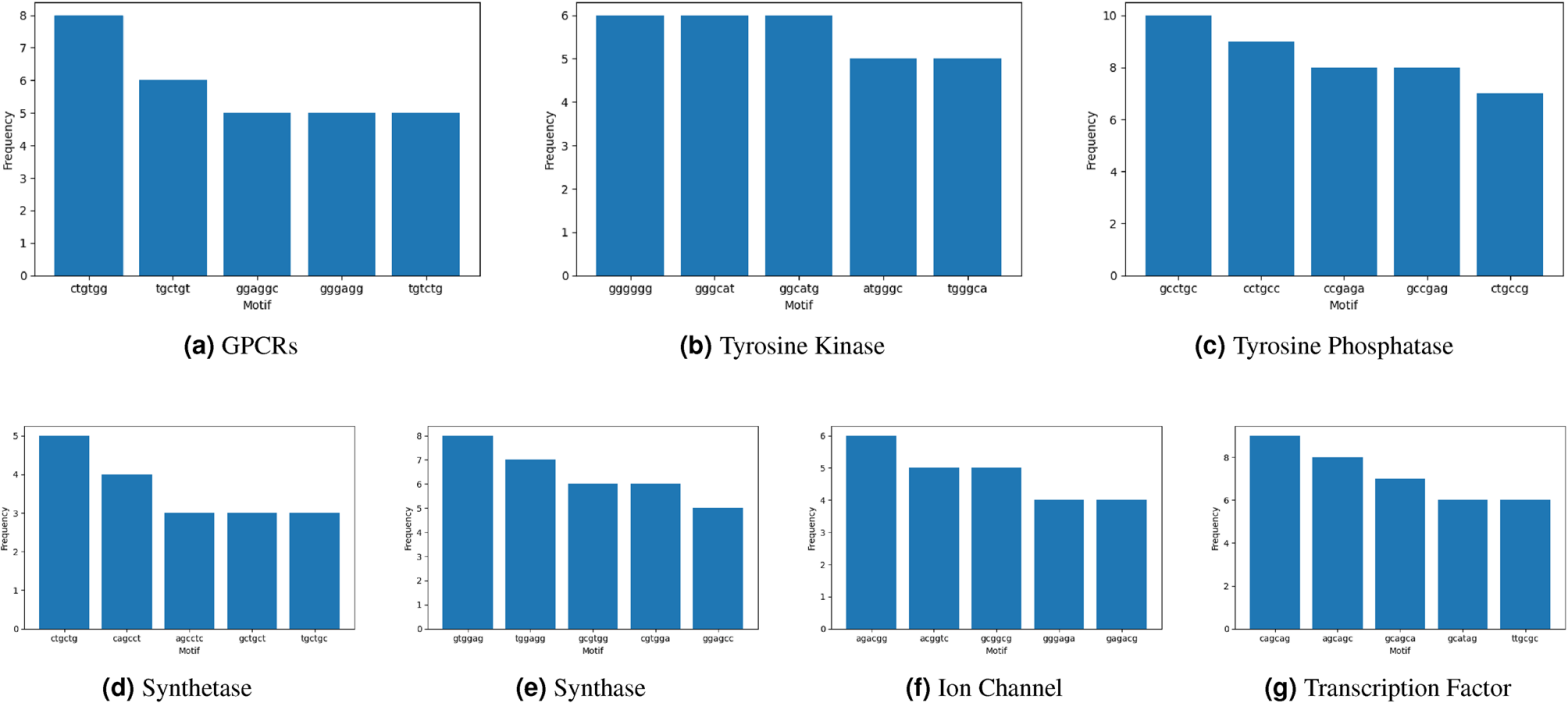
Consensus motif frequency across models and XAI methods. Each subplot displays the occurrence of the top motifs detected for a specific functional group, aggregated across Saliency, GradientSHAP, Integrated Gradients and Attention analyses.

Figure 11 summarizes the consensus frequency of motifs identified by all XAI methods and models. This combined view highlights recurring motif patterns that were consistently detected across explainability techniques and learning approaches for each functional group.

Across all functional groups, the XAI analysis focused on identifying consensus sequence motifs that were consistently detected across machine learning, deep learning, and multiple interpretability methods. These motifs represent biologically plausible sequence signatures aligned with known structural, catalytic, or regulatory features of the corresponding protein families^48^.

For GPCRs, motifs such as ctgtgg, tgctgt, and ggaggc corresponded to conserved transmembrane and cytoplasmic elements, including CWxP-related residues and glycine-rich hinge regions involved in receptor activation. Motif comparison using the Tomtom tool against the JASPAR2020 Conserved Non-coding Elements (CNE) database revealed multiple significant matches (e.g., ctgtgg: 16 matches, tgctgt: 29 matches, ggaggc: 11 matches), supporting the biological relevance and conservation of these motifs across regulatory sequence databases.

In Tyrosine Kinases, glycine-enriched motifs resembling the canonical GxGxxG P-loop were repeatedly identified, indicating that the models captured ATP-binding and catalytic-site signatures. This observation was further supported by motif comparison, where motifs such as gggcat and ggcatg exhibited multiple matches (8 and 13 matches, respectively), while highly repetitive motifs like gggggg showed no significant matches. This suggests that structured glycine-containing patterns, rather than simple homopolymeric repeats, are more biologically relevant and align with known regulatory motif databases.

For Tyrosine Phosphatases, motifs such as gcctgc and cctgcc aligned with patterns surrounding the HCX$_5$R catalytic motif, reflecting recognition of conserved dephosphorylation domains. This was further supported by motif comparison, where gcctgc and cctgcc showed multiple matches (13 and 21 matches, respectively), while less frequent motifs such as ccgaga exhibited limited matches. These results reinforce the biological relevance of the identified motifs and their association with conserved functional regions.

In Synthetases, recurring motifs including ctgctg and cagcct reflected hydrophobic and flexible residue patterns associated with conserved aminoacylation scaffolds. Motif comparison further supported this observation, with ctgctg showing a high number of matches (31), followed by cagcct (14 matches), while related motifs such as agcctc exhibited fewer matches (7). These results indicate that the identified motifs correspond to biologically conserved sequence patterns relevant to synthetase function.

Synthase motifs such as gtggag and tggagg corresponded to short structural segments linked to substrate binding and catalytic turnover. Motif comparison revealed limited but notable matches, with tggagg showing multiple matches (7) and gtggag exhibiting a single match, while motifs such as gcgtgg showed no significant matches. These findings suggest that specific structured motifs are biologically relevant, whereas others may represent weaker or less conserved patterns.

For Ion Channels, GC-rich motifs such as gcggcg and agacgg resembled promoter and enhancer elements characteristic of ion-channel gene regulation. Motif comparison revealed that gcggcg exhibited multiple matches (10) to GC-binding transcription factors such as KLF family members and ZNF proteins, while agacgg matched neurodevelopmental regulators including Atoh1, NEUROG2, and NEUROD family proteins (5 matches). Additional motifs such as acggtc also showed relevant matches (4), further supporting the regulatory significance of these patterns. These findings indicate that the identified motifs align with known transcription factor binding sites involved in ion-channel gene expression and regulation.

Finally, in Transcription Factors, consensus motifs including cagcag and agcagc reflected compositional patterns associated with activation domains and CpG-rich promoter elements, indicating that the framework effectively captured regulatory and domain-specific sequence signatures. Motif comparison revealed strong conservation, with cagcag and agcagc exhibiting a high number of matches (31 and 26 matches, respectively), along with related motifs such as gcagca also showing substantial correspondence. These findings confirm that the identified motifs align with conserved regulatory elements commonly associated with transcription factor binding and promoter activity.

#### Cross-species motif comparison

**Table 9:** Cross-species comparison of the top 10 motifs identified for GPCR functional group using Feature Importance on Logistic Regression (LR) and Integrated Gradients on CNN for each dataset. The corresponding Feature Importance and Integrated Gradients scores are listed alongside each motif.

Species	LR feature importance (Top-10 Motifs of GPCR)	CNN integrated gradients (Top-10 Motifs of GPCR)
Human	tcgctg (0.2166), gtcctg (0.2010), aacaac (0.1985), agccag (0.1940), gctgtg (0.1936), cagcca (0.1933), tgctct (0.1840), cttctg (0.1839), tgcggc (0.1799), ttttca (0.1752)	tgttcc (21.4962), cctgct (19.4643), ctgctc (19.3442), ctcttc (19.3343), tgctct (18.6367), ttcctg (18.2247), gttcct (16.3187), cttctg (16.1633), ctgctg (14.4151), tcttct (12.9497)
Chimpanzee	gctgtg (0.2060), gcttcc (0.2033), tcatct (0.2020), tcttca (0.1904), catcat (0.1787), tcgtgg (0.1752), ccctgg (0.1748), tgacca (0.1717), cctggg (0.1709), tgcctg (0.1679)	cctgct (10.4112), ctgctc (10.2236), tgctct (6.7357), ctcttc (5.6141), ccctgc (5.4651), ctcctg (4.5408), tgctcc (4.3208), ctgctg (4.1764), gctcct (3.7282), ctggcc (3.5170)
Dog	cctgct (0.2274), ctctac (0.2260), acctgg (0.2252), ttcatt (0.2086), cctggc (0.2061), ctgctc (0.2004), tcttct (0.1834), cctggg (0.1779), tcatct (0.1687), ttgttt (0.1659)	gctgct (3.7453), ctgctc (3.4347), tcctgg (3.0909), tgctgc (3.0881), cctgct (2.3990), ctgctg (2.3687), cttcct (2.3247), ctgcct (2.2193), tgcctc (2.2141), ttcctg (2.0479)

Table 9 presents the cross-species comparison of the top 10 motifs identified for the GPCR functional group using Feature Importance on Logistic Regression and Integrated Gradients on CNN. It is important to note that this analysis represents a comparative study of motif patterns across species, rather than a strict cross-species generalization setting where models are trained on one species and evaluated on another. Feature Importance and Integrated Gradients were used to extract the motifs for LR and CNN, respectively.

For Logistic Regression, no motif was consistently present across all three species, reflecting the species-specific variability in GPCR sequence composition. However, several motifs such as cctggg, gctgtg, and tcatct appeared in at least two species. Motif comparison using the Tomtom tool against the JASPAR2020 CNE database revealed that these motifs exhibited moderate levels of conservation (cctggg: 14 matches, gctgtg: 29 matches, tcatct: 23 matches), supporting their partial biological relevance despite cross-species variability. These motifs correspond to short k-mer patterns enriched in GC-rich or mixed GC–AT regions, which may loosely relate to conserved segments surrounding transmembrane helices or cytoplasmic loop regions in GPCRs, where variability across species is common but partial conservation can still arise.

For CNN, clear consensus motifs emerged across species, including ctgctg, ctgctc, and cctgct. Additional motifs occurring in at least two species, such as tgctct, ttcctg, and ctcttc, suggest that the convolutional filters consistently captured recurring structural sequence fragments. Importantly, these motifs showed strong validation, with highly repetitive core patterns such as ctgct and its variants demonstrating extensive matches (up to 36 for ctgct, 31 for ctgctg, and 21 for cctgct), while supporting motifs like ttcctg and ctcttc also exhibited substantial correspondence. These motifs exhibit strong internal repetitiveness (for example, ctgct), which CNNs are well-suited to detect because convolutional kernels naturally emphasize spatially local, repeatedly occurring k-mer patterns.

Comparing the two approaches, LR primarily highlighted motifs with species-specific discriminatory value, while CNN extracted more stable, recurrent sequence motifs across species. This difference reflects the underlying model architectures: LR relies on linear feature–class associations specific to each dataset, whereas CNN’s hierarchical filters learn generalizable local patterns that are more likely to recur across species. The stronger database alignment observed for CNN-derived motifs further reinforces their biological plausibility and cross-species consistency.

#### Cross-model motif analysis

**Table 10:** Cross-model XAI comparison on the Human dataset. Feature Importance is applied to Logistic Regression, Random Forest, and Multinomial Naive Bayes to identify the top 10 motifs for the Synthase functional group, while Integrated Gradients is applied to CNN, CNN–Attention, and CNN–BiLSTM to extract the top 10 motifs for the Transcription Factor functional group.

Model type	Human (Top-10 Motifs + Scores)
Logistic regression (synthase)	ctggag (0.2636), tgtccg (0.2563), acattg (0.2516), gtgggg (0.2501), ctgtgc (0.2411), gacaga (0.2388), ggttct (0.2360), tggggc (0.2260), ttggaa (0.2254), ggtact (0.2212)
Random forest (synthase)	cagcag (0.0029), agcagc (0.0020), tgctgg (0.0019), ggcatg (0.0018), ctggtg (0.0018), ggatgg (0.0017), ccagca (0.0017), tggtgt (0.0015), gtgctg (0.0015), ctctac (0.0015)
Multinomial Naive Bayes (synthase)	ctggag (− 6.4281), ctgctg (− 6.5712), ctgcag (− 6.6703), cctgga (− 6.6816), tggtgg (− 6.6930), tcctgg (− 6.7029), tgctgg (− 6.7112), gctgga (− 6.7348), cagctg (− 6.7643), gctgct (− 6.7875)
CNN (transcription factor)	cagcag (76.8405), agcagc (53.1437), gcagca (32.3086), aggagg (25.3584), ggagga (23.4207), agcccc (22.3878), ccagca (21.2277), ccccgg (20.1202), gcggcg (19.8683), agcaga (19.0683)
CNN–attention (transcription factor)	cagcag (171.9802), agcagc (101.3025), ccagca (77.1736), gcagca (67.2537), aggagg (58.4936), cacaga (54.7885), cggagg (44.4612), agctca (40.4150), ctggaa (37.5330), tggagg (35.8858)
CNN–BiLSTM (transcription factor)	agcagc (41.4212), atggcc (29.7714), cagcag (23.7486), atggac (21.9047), gcagca (21.3839), tggagg (15.7186), atggcg (13.0852), agaaga (12.0084), gcccga (11.2745), aaagaa (10.9821)

Table 10 enables a cross-model comparison of the motifs identified by ML and DL models on the Human dataset. For the ML models (Logistic Regression, Random Forest, and Multinomial Naive Bayes), no strict consensus motif appeared across all three models, which shows that each algorithm relied on different discriminatory k-mers for classifying Synthase sequences. A small majority consensus was observed, where the motifs ctggag and tgctgg were shared by at least two ML models. Motif comparison using the Tomtom tool against the JASPAR2020 CNE database revealed that these motifs exhibit moderate conservation (ctggag: 14 matches, tgctgg: 22 matches), supporting their biological relevance. These motifs correspond to compact GC-rich segments that have been associated with short residue pairs or hinge-like loop regions involved in substrate positioning, catalytic turnover, or conformational gating in synthases.

The DL models showed much stronger agreement. The motifs agcagc, cagcag, and gcagca were identified by all three deep learning architectures (CNN, CNN Attention, and CNN BiLSTM), indicating consistent detection of conserved sequence fragments. The majority consensus motifs, which include agcagc, aggagg, cagcag, ccagca, gcagca, and tggagg, align with transcription factor signatures such as poly-glutamine, poly-serine, and poly-alanine tracts, as well as sequence patterns known to occur in activation or repression domains. Importantly, these motifs showed strong validation, with cagcag and gcagca demonstrating extensive matches (31 each), agcagc showing 26 matches, and supporting motifs such as ccagca (22 matches) and aggagg (8 matches) further reinforcing their biological significance. This consistent and high level of correspondence indicates that the DL models captured highly conserved regulatory sequence patterns.

When comparing ML and DL behaviour, ML models tended to highlight motifs that are specific to the Synthase class and that differ between algorithms, while the DL architectures consistently identified stable and biologically interpretable transcription factor motifs. This difference reflects the inductive biases of the two model families, where ML models rely on linear feature associations and DL models learn hierarchical sequence representations that capture shared regulatory or structural patterns with greater consistency. The stronger database alignment observed for DL-derived motifs further reinforces their biological plausibility and robustness.

#### Stability analysis of motifs

**Table 11:** Stability analysis across all functional groups on the Combined dataset. For each ML or DL model pair, the Jaccard index and overlap percentage were computed based on the top 10 identified motifs.

Functional group	ML stability			DL Stability		
	Model pair	Jaccard	Overlap %	Model pair	Jaccard	Overlap %
GPCR	LR–RF	0.000	0%	CNN–CNN-Attention	0.250	40%
	LR–MNB	0.000	0%	CNN–CNN-BiLSTM	0.429	60%
	RF–MNB	0.176	30%	CNN-Attention–CNN-BiLSTM	0.111	20%
Tyrosine kinase	LR–RF	0.111	20%	CNN–CNN-Attention	0.111	20%
	LR–MNB	0.000	0%	CNN–CNN-BiLSTM	0.176	30%
	RF–MNB	0.176	30%	CNN-Attention–CNN-BiLSTM	0.111	20%
Tyrosine phosphatase	LR–RF	0.000	0%	CNN–CNN-Attention	0.053	10%
	LR–MNB	0.000	0%	CNN–CNN-BiLSTM	0.176	30%
	RF–MNB	0.053	10%	CNN-Attention–CNN-BiLSTM	0.000	0%
Synthetase	LR–RF	0.000	0%	CNN–CNN-Attention	0.111	20%
	LR–MNB	0.000	0%	CNN–CNN-BiLSTM	0.053	10%
	RF–MNB	0.053	10%	CNN-Attention–CNN-BiLSTM	0.000	0%
Synthase	LR–RF	0.000	0%	CNN–CNN-Attention	0.053	10%
	LR–MNB	0.053	10%	CNN–CNN-BiLSTM	0.176	30%
	RF–MNB	0.111	20%	CNN-Attention–CNN-BiLSTM	0.000	0%
Ion channel	LR–RF	0.000	0%	CNN–CNN-Attention	0.000	0%
	LR–MNB	0.000	0%	CNN–CNN-BiLSTM	0.429	60%
	RF–MNB	0.111	20%	CNN-Attention–CNN-BiLSTM	0.000	0%
Transcription factor	LR–RF	0.000	0%	CNN–CNN-Attention	0.176	30%
	LR–MNB	0.000	0%	CNN–CNN-BiLSTM	0.250	40%
	RF–MNB	0.176	30%	CNN-Attention–CNN-BiLSTM	0.333	50%

Table 11 shows clear differences in motif stability between the ML and DL models. Across all functional groups, ML models exhibited very low stability, with Jaccard values mostly at 0.000 and a peak overlap of only 30% for RF–MNB in GPCR, Tyrosine Kinase, and Transcription Factor, which indicates that the linear and tree-based models relied on highly model-specific k-mers. In contrast, the DL models demonstrated substantially higher consistency, with peak overlap values reaching 60% for CNN–CNN-BiLSTM in both GPCR and Ion Channel, and Jaccard scores up to 0.429. This pattern reflects the architectural strengths of DL models, which learn hierarchical and spatially local sequence patterns that generalize across architectures, whereas ML models depend heavily on dataset-specific linear or probabilistic splits that seldom align across models. Overall, the stability results show that DL models provide more reproducible and biologically coherent motif discovery than ML models.

#### Fidelity analysis of motifs

**Table 12:** Fidelity analysis across seven functional groups using ML and DL models. Values represent the absolute decrease in accuracy, F1-score, and MCC after masking the motifs identified by each model.

FG	LR			RF			MNB			CNN			CNN–Attention			CNN–BiLSTM		
	Acc	F1	MCC	Acc	F1	MCC	Acc	F1	MCC	Acc	F1	MCC	Acc	F1	MCC	Acc	F1	MCC
GPCR	0.0036	0.0037	0.0045	0.0145	0.0161	0.0172	0.0523	0.0730	0.0648	0.0022	0.0019	0.0026	0.0312	0.0342	0.0383	0.0029	0.0024	0.0035
Tyrosine Kinase	0.0153	0.0152	0.0186	0.0145	0.0161	0.0172	0.0763	0.0873	0.0863	0.0029	0.0004	0.0035	0.0189	0.0205	0.0231	0.0022	0.0014	0.0027
Tyrosine Phosphatase	0.0094	0.0125	0.0116	0.0145	0.0161	0.0172	0.1082	0.1174	0.1179	0.0044	0.0034	0.0053	0.0312	0.0360	0.0380	0.0007	0.0013	0.0009
Synthetase	0.0087	0.0098	0.0108	0.0145	0.0161	0.0172	0.0617	0.0833	0.0745	0.0036	0.0028	0.0044	0.0545	0.0534	0.0659	0.0015	0.0012	0.0018
Synthase	0.0080	0.0073	0.0098	0.0145	0.0161	0.0172	0.0508	0.0750	0.0631	0.0000	0.0005	0.0000	0.0530	0.0544	0.0649	0.0029	0.0030	0.0035
Ion Channel	0.0065	0.0144	0.0081	0.0145	0.0161	0.0172	0.0668	0.0780	0.0768	0.0065	0.0059	0.0079	0.0203	0.0209	0.0248	0.0022	0.0021	0.0026
Transcription Factor	0.0167	0.0143	0.0198	0.0145	0.0161	0.0172	0.1089	0.1071	0.1157	0.0087	0.0056	0.0105	0.0436	0.0422	0.0525	0.0015	0.0015	0.0018

Table 12 presents the fidelity analysis, which measures how much model performance deteriorates when the motifs identified by each model are masked. Across all functional groups, Multinomial Naive Bayes displays the largest absolute drops in accuracy, F1-score, and MCC, often exceeding 0.10 for Tyrosine Phosphatase and Transcription Factor. This indicates that MNB relies heavily on specific k-mer frequency patterns, and removing these motifs disrupts its probabilistic assumptions. Random Forest also shows moderate but consistent performance loss, suggesting that the decision trees depend on these high-importance motifs for splitting.

In contrast, Logistic Regression and deep learning models such as CNN, CNN–Attention, and CNN–BiLSTM exhibit comparatively small performance drops across most functional groups. One possible interpretation is that these models learn more distributed and redundant feature representations, making them less sensitive to the removal of any single motif.

At the same time, the consistently low degradation, which is often below 1–2%, suggests that motif-level explanations capture only part of the decision-making process in these models, where predictions may be influenced by combinations of features rather than isolated patterns. This behavior reflects the inherent difference in how simpler and more expressive models encode information.

Therefore, the fidelity results highlight an important trade-off. While ML models such as MNB show higher interpretability with direct dependence on identifiable motifs, more complex models demonstrate greater robustness due to their reliance on distributed representations. This observation emphasizes that fidelity-based evaluation should be interpreted in the context of model architecture and representation learning, particularly for sequence-based tasks.

## Conclusion

This study introduced an explainable AI framework that integrates machine learning and deep learning models for multi-species DNA functional group classification. Using k-mer representations and controlled hyperparameter tuning, Logistic Regression achieved the strongest overall performance, outperforming deep learning models and previously reported results on the same datasets. This highlights that, for high-dimensional sparse k-mer features and moderate dataset sizes, classical linear models remain highly effective and can outperform more complex architectures.

While deep learning models did not provide consistent improvements in predictive performance, they contributed to the framework by capturing localized and contextual motif patterns, which were further analyzed using multi-level XAI techniques. The integration of Feature Importance, Saliency Maps, Integrated Gradients, GradientSHAP, and Attention Heatmaps enabled the extraction of consensus motifs, stability patterns, and fidelity behaviour, revealing biologically meaningful sequence signatures across datasets.

The main limitations of this work include the use of a fixed k-mer representation, which introduces a bias toward capturing local sequence patterns while limiting the ability to model long-range dependencies and higher-order structural interactions in DNA sequences. As a result, important regulatory relationships spanning larger genomic regions may not be fully captured. In addition, the moderate dataset size impacts the effectiveness of deep learning models, which typically require larger datasets to learn robust and generalizable representations.

From a scalability perspective, the k-mer-based approach leads to exponentially growing feature dimensionality with increasing k and dataset diversity, which can significantly increase computational complexity and memory requirements, particularly for machine learning models using sparse representations. Similarly, extending the framework to larger multi-species genomic datasets would require careful consideration of model capacity, training efficiency, and data heterogeneity.

Future work will focus on exploring transformer-based representations to better capture long-range dependencies in genomic sequences, as well as scaling the framework to larger and more diverse datasets. Incorporating experimentally validated annotations will further enhance the biological interpretability of the discovered motifs. Additionally, extending the framework to evaluate cross-species generalization through transfer-based settings, such as training on one species and testing on another, represents an important direction for assessing the robustness and broader applicability of the proposed approach.

## Data Availability

Original data generated and analyzed during this study are included in the Kaggle repository, DNA Sequence Dataset, which is also listed in References.

## Code availability

Original code generated during this study is included in the GitHub repository, DNA Research Paper.
